# The association of insulin resistance and carotid atherosclerosis with thigh and calf circumference in patients with type 2 diabetes

**DOI:** 10.1186/1475-2840-11-62

**Published:** 2012-06-08

**Authors:** Jong Suk Park, Min Ho Cho, Chul Woo Ahn, Kyung Rae Kim, Kap Bum Huh

**Affiliations:** 1 Division of Endocrinology, Department of Internal Medicine, Yonsei University College of Medicine, Seoul, South Korea; 2 Severance Institute for Vascular and Metabolic Research, Yonsei University College of Medicine, Seoul, South Korea; 3 Huh’s Diabetes Center and the 21st Century Diabetes and Vascular Research Institute, Seoul, South Korea

**Keywords:** Insulin resistance, Intima media thickness, Thigh circumference, Calf circumference, Type 2 diabetes mellitus

## Abstract

**Background:**

The relationship between body composition parameters such as thigh and calf circumference and insulin resistance or atherosclerosis in type 2 diabetes is poorly understood. The aim of this study was to investigate the relationship between insulin resistance, atherosclerosis, and thigh and calf circumference in patients with type 2 diabetes.

**Methods:**

A total of 4,427 subjects with type 2 diabetes were enrolled in this study. Insulin sensitivity was assessed according the rate constant for plasma glucose disappearance (Kitt) determined via the short insulin tolerance test. Biochemical and anthropometric profiles were measured according to a standardized protocol. Visceral fat thickness and carotid intima media thickness (IMT) were measured by ultrasonography.

**Results:**

Insulin sensitivity index (Kitt) was significantly correlated with weight adjusted thigh and calf circumference. Thigh circumference was inversely associated with IMT in men and women and calf circumference was negatively correlated with IMT in women. Multiple stepwise regression analysis revealed that thigh circumference was independently correlated with insulin sensitivity index (Kitt) and IMT. Furthermore, in multivariate logistic regression analysis, thigh circumference was an independent determinant factor for carotid atherosclerosis in patients with type 2 diabetes even after adjusting for other cardiovascular risk factors.

**Conclusions:**

Thigh and calf circumference were correlated with insulin resistance and carotid atherosclerosis, and thigh circumference was independently associated with insulin resistance and carotid atherosclerosis in patients with type 2 diabetes.

## Introduction

Insulin resistance is associated with increased risk of cardiovascular disease (CVD), hypertension, obesity and dyslipidemia and is related to the development of type 2 diabetes [1]. Obesity, especially abdominal obesity, may play a major role in insulin resistance and various metabolic risk factors [2-6], however, recent studies have suggested that body muscle mass is also related to insulin resistance [1,7]. As skeletal muscle is an important site of glucose uptake and deposition [8], loss of muscle mass could influence insulin sensitivity and promote metabolic disorders such as diabetes mellitus [1]. Moreover recently, peripheral adiposity in lower body was found to be associated with insulin resistance and glucose metabolism [9,10]. Thigh circumference and calf circumference reflect body muscle mass and peripheral subcutaneous fat [11,12]. However, there are few reports on the relationship between insulin resistance and thigh and calf circumference [13].

CVD is one of the most common causes of mortality in patients with type 2 diabetes, and carotid atherosclerosis determined by intima media thickness (IMT) is used to predict CVD and related outcomes [14-19]. The association of carotid IMT with body composition parameters and fat distribution has not been fully investigated. Several studies have demonstrated a correlation between carotid IMT and abdominal obesity [20-22], but there are few studies of the relationship between body composition parameters such as thigh and calf circumference and carotid atherosclerosis [23,24]. Therefore, in the present study, we investigated the relationship between insulin resistance, IMT, and thigh and calf circumference in patients with type 2 diabetes.

## Methods

### Study population

From 2002 to 2009, total 9,894 patients with type 2 diabetes participated in the Korean Metabolic Syndrome Research Initiative at Huh’s Diabetes Center and Diabetes Clinic of Kangnam Severance Hospital. Diagnosis of type 2 diabetes mellitus was based on a previous history of diabetes mellitus or criteria from the American Diabetes Association’s diagnostic guidelines. Subjects who had been measured thigh and calf circumference and assessed for both insulin sensitivity and IMT were included in the study. We excluded subjects with a concurrent acute illness including clinically significant infectious disease and chronic disease such as malnutrition, hepatic disease, renal failure and malignant disease. We also excluded subjects with a history of cardiovascular or cerebrovascular disease. Patients who had received thiazolidinedione and insulin were also excluded, as it has been reported to affect insulin resistance as well as IMT. A total of 4,427 subjects were included in the final analyses. The Institutional Review Board of Yonsei University College of Medicine approved the study protocol, and written informed consent was obtained from all participants.

### Anthropometric and biochemical parameters

Height, weight and waist circumference (WC) were measured, and body mass index (BMI) was calculated by dividing weight (kg) by the square of height (m^2^). WC was measured at the midpoint between the lower border of the rib cage and the iliac crest. Thigh circumference was measured at the midpoint from the inguinal crease to the proximal pole of the patella, and calf circumference was measured at the point of greatest circumference. Visceral fat thickness (VFT) was measured using a high resolution ultrasonographic system with a 3.5-MHz convex probe (OGIQ 7, GE, Milwaukee, WI, USA). VFT was defined as the distance between the anterior wall of the aorta and the internal face of the rectoabdominal muscle perpendicular to the aorta at the point of 1 cm above the umbilicus [25]. We measured blood pressure (BP) in the right upper arm of patients in a sedentary posture while they were seated after having rested for at least 5 min using a standard sphygmomanometer or an automatic oscillometric BP recorder.

### Biochemical parameters

Blood samples were taken from all subjects after eight hours of fasting. Samples were immediately centrifuged, and plasma and serum samples were stored at −70°C until analysis. Glucose was measured by a standard glucose oxidase method (747 Automatic Analyzer, Hitachi, Tokyo, Japan). Total cholesterol (TC), high density lipoprotein cholesterol (HDL-C), and triglycerides (TG) were measured enzymatically using a chemical analyzer (Daiichi, Hitachi 747, Japan). Subjects with TG levels more than 400 mg/dL not included and low density lipoprotein cholesterol (LDL-C) was calculated according to the Friedewald formula. Fasting serum insulin was determined by chemiluminescence (RIA Kit, Daiichi, Japan) and glycosylated hemoglobin was measured by immunoturbidimetry (Cobas Integra 800, Roche, Mannheim, Germany), and insulin resistance was estimated using the Homeostasis Model Assessment of Insulin Resistance (HOMA-IR) index, calculated from the following formula: $HOMA-IR=fasting\;insulin\;\left(\mu U/mL\right)\times fasting\;plasma\;glu\cos e\;\left(mmol/L\right)/22.5$

### Assessment of insulin sensitivity

To evaluate insulin resistance in all subjects, a short insulin tolerance test was performed and assessed by insulin sensitivity index (Kitt) [26-29]. After 12 h of fasting, an 18-gauge catheter was inserted into one arm, a three-way was connected, and blood was obtained. In the other arm, an antecubital vein was secured for injecting an insulin and dextrose solution. Insulin that had been diluted 100 times (insulin lispro, 0.1 u/kg) was then injected. Blood was obtained through the three-way just prior to injection (at “0 min”), and at 3, 6, 9, 12, and 15 min after the injection, serum glucose was measured, and the Kitt value was obtained using the following formula:

(1) $$Kitt\left(rate\;cons\tan t\;for\;plasma\;glu\cos e\;disappearance\right)=0.693/t1/2*100\left(\%/\min\right)$$

### Carotid artery intima media thickness (IMT)

Carotid IMT was evaluated by high-resolution B-mode ultrasonography on a single machine (OGIQ 7, GE, Milwaukee, WI, USA) with a 7.5-MHz linear array transducer. Intima media thickness was the distance between the lumen intima interface and media adventitia interface. Measurements of carotid IMT were conducted at three differential plaque free sites: the site of greatest thickness and 1 cm upstream and 1 cm downstream from the site of greatest thickness. The mean of the three measurements of the right and left IMT was defined as the mean IMT. Carotid atherosclerosis was defined as mean IMT ≥ 1.0 mm, as assessed by B-mode ultrasound [30-33].

### Statistical analysis

Data are expressed as means ± S.D. Intergroup comparisons were performed using ANOVA. Because of the skewed distribution, insulin and TG values were presented as the median (interquartile range) and were log transformed for analysis. A Pearson’s correlation analysis was also performed to evaluate the relationship between insulin resistance, IMT and clinical factors including thigh and calf circumference, where indicated. Stepwise multiple linear regression analysis was performed to analyze independent association of insulin sensitivity index (kitt) and IMT adjusting for confounding factors. We selected all the variables shown to be related to kitt and IMT in simple correlation analysis including age, BMI, blood pressure, lipid, smoking habits, and treatment medications. The odds ratios (ORs) with 95% confidence intervals (CIs) predicting carotid atherosclerosis by thigh and calf circumference were determined by multivariate logistic regression analyses after controlling for other potential confounders. Statistical analyses were carried out using SPSS for Windows 15.0 (SPSS Inc., Chicago, IL, USA). P-values less than 0.05 were considered statistically significant.

## Results

**Figure 1:**
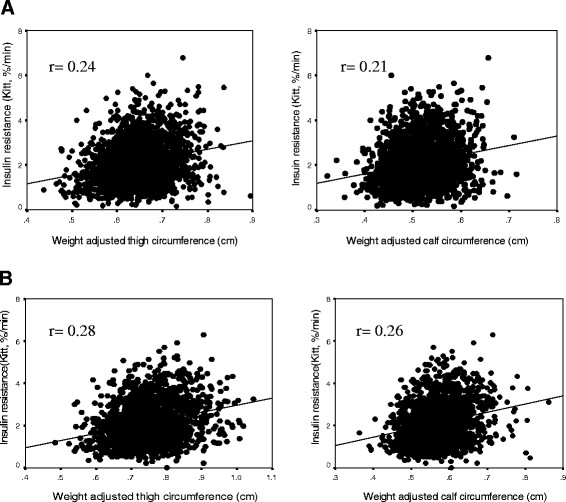
**The relationship between insulin resistance and thigh and calf circumference in men (A) and women (B).** The insulin sensitivity index (Kitt) was significantly correlated with weight adjusted thigh and calf circumference in both men and women (P < 0.01).

**Figure 2:**
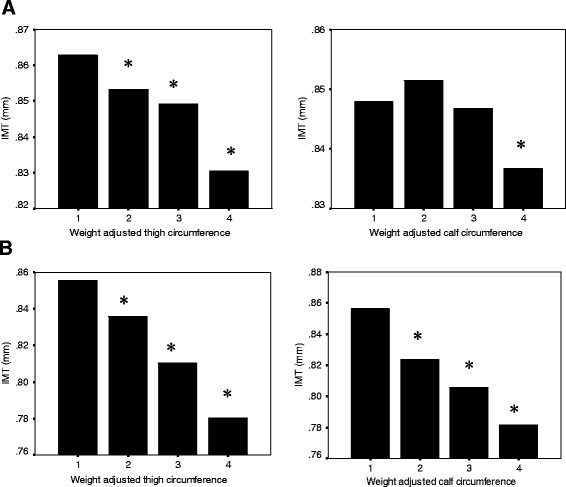
**The relationship between intima media thickness and thigh and calf circumference in men (A) and women (B).** Carotid IMT differed among the four groups of thigh and calf circumference (P <0.05 vs. first tertile)

The clinical and laboratory characteristics of the study population are summarized in Table 1. The study included 2,323 men aged 55.75 ± 11.63 years and 2,104 women aged 56.50 ± 11.67 years. Thigh and calf circumference were adjusted for body weight and height, and since thigh and calf circumference showed a stronger association with body weight (thigh circumference, calf circumference: r = 0.701, r = 0.729 in men, r = 0.722, r = 0.744 in women; all p < 0.01) than body height (thigh circumference, calf circumference: r = 0.280, r = 0.327 in men, r = 0.171, r = 0.368 in women; all p < 0.01), they were adjusted by body weight (Additional file 1: Figure S 1, S 2). The insulin sensitivity index (Kitt) was significantly correlated with weight adjusted thigh and calf circumference in both men and women (Table 2, Figure 1). Figure 2 shows carotid IMT in quartiles of thigh and calf circumference. In both men and women, carotid IMT differed among the four groups of thigh and calf circumference. Thigh circumference was inversely associated with IMT in men and women, and calf circumference was negatively correlated with IMT in women (Table 2).

**Table 1:** Baseline clinical characteristics of subjects

	**Men**	**Women**
N	2,323	2,104
Age (years)	55.75 ± 11.63	56.50 ± 11.67
Duration of diabetes (years)	6.67 ± 6.99	6.54 ± 6.20
SBP (mmHg)	134.02 ± 17.03	136.84 ± 18.87
DBP (mmHg)	87.86 ± 11.25	85.28 ± 11.32
Weight (kg)	70.89 ± 10.20	59.34 ± 9.08
BMI (kg/m^2^)	24.57 ± 3.25	24.56 ± 3.34
WC (cm)	86.23 ± 7.74	79.41 ± 8.31
Smoking (n, %)	354 (15.2%)	32 (1.5%)
Antihypertensive medication, N (%)	720 (30.9)	694 (32.9)
Antidyslipidemic medication, N (%)	464 (19.9)	487 (23.1)
Antidiabetic medication		
Sulfonylureas	1441 (62.0)	1328 (63.1)
Metformin	973 (41.8)	1032 (49.0)
AGIs	246 (10.6)	231 (10.9)
FPG (mmol/L)	8.25 ± 2.69	7.91 ± 2.69
TC (mmol/L)	4.99 ± 1.02	5.27 ± 1.07
TG (mmol/L)	1.55 (1.00-3.19)	1.38 (1.00-2.42)
LDL-C (mmol/L)	2.96 ± 0.88	3.16 ± 0.96
HDL-C (mmol/L)	1.23 ± 0.30	1.35 ± 0.34
Insulin (μIU/mL)	6.9 (5.1-9.4)	7.6 (5.6-10.2)
HOMA-IR	2.79 ± 1.73	2.94 ± 1.97
HbA1c (%)	8.37 ± 2.00	8.32 ± 1.85
VAT (mm)	48.39 ± 17.23	39.94 ± 16.45
Calf circumference (mm)	35.96 ± 3.19	33.22 ± 2.64
Thigh circumference (mm)	45.19 ± 4.03	43.88 ± 3.99
Mean IMT(mm)	0.83 ± 0.19	0.80 ± 0.18
Kitt (%/min)	2.28 ± 0.96	2.31 ± 0.94

Data are mean ± SD or proportion (%). Data for TG and insulin were skewed distribution, and were presented as the median (interquartile range). SBP, systolic blood pressure; DBP, diastolic blood pressure; BMI, body mass index; WC, waist circumference; FPG, fasting plasma glucose; TC, total cholesterol; TG, triglyceride; LDL-C, low-density lipoprotein cholesterol; HDL-C, high-density lipoprotein cholesterol; Homeostasis Model Assessment of Insulin Resistance, HOMA-IR; HbA1c, hemoglobin A1c; VAT, visceral adipose thickness; IMT, intima media thickness; Kitt, rate constant for plasma glucose disappearance.

**Table 2:** Correlation between insulin sensitivity index (Kitt), IMT and other variables

	**Insulin sensitivity 1index (Kitt)**		**IMT**	
	**Men**	**Women**	**Men**	**Women**
Age	−0.051^**^	−0.139^**^	0.410^**^	0.369^**^
Duration of diabetes	−0.064^**^	−0.060^*^	0.212^**^	0.147^**^
SBP	−0.125^**^	−0.164^**^	0.196^**^	0.220^**^
DBP	−0.126^**^	−0.104^**^	0.005	0.039^*^
Weight	−0.159^**^	−0.192^**^	0.064^**^	0.044^*^
BMI	−0.196^**^	−0.216^**^	0.029	0.064^**^
WC	−0.277^**^	−0.300^**^	0.063^**^	0.100^**^
Smoking habits	−0.038	0.017	0.034	0.009
FPG	−0.351^**^	−0.370^**^	0.025	0.006
TC	−0.140^**^	−0.138^**^	0.042^*^	0.071^**^
TG	−0.276^**^	−0.294^**^	0.041^*^	0.074^*^
LDL-C	−0.052^**^	−0.036^*^	0.080^**^	0.071^**^
HDL-C	0.103^**^	0.112^**^	−0.024^*^	−0.037^*^
Insulin	−0.215^**^	−0.218^**^	0.022	0.072^**^
HOMA-IR	−0.347^**^	−0.367^**^	0.050^**^	0.053^**^
HbA1c	−0.286^**^	−0.195^**^	0.073^**^	0.016
VAT	−0.284^**^	−0.313^**^	0.057^**^	0.102^**^
Weight adjusted calf circumference	0.211^**^	0.258^**^	−0.027	−0.173^**^
Weight adjusted thigh circumference	0.244^**^	0.279^**^	−0.102^*^	−0.170^**^
Mean IMT	−0.062^**^	−0.092^**^		
Kitt			−0.062^**^	−0.092^**^

* P < 0.05, **P <0.01.

SBP, systolic blood pressure; DBP, diastolic blood pressure; BMI, body mass index; WC, waist circumference; FPG, fasting plasma glucose; TC, total cholesterol; TG, triglyceride; LDL-C, low-density lipoprotein cholesterol; HDL-C, high-density lipoprotein cholesterol; Homeostasis Model Assessment of Insulin Resistance, HOMA-IR; HbA1c, hemoglobin A1c; VAT, visceral adipose thickness; IMT, intima media thickness; Kitt, rate constant for plasma glucose disappearance.

In stepwise multiple linear regression analysis, WC, FPG, TG, HbA1c, insulin, VAT and thigh circumference were significantly associated with insulin sensitivity index (kitt), but age, blood pressure, calf circumference and smoking habits, and treatment medications were not associated with insulin sensitivity index (kitt) (Table 3). Stepwise multiple linear regression analysis for IMT showed that thigh circumference was independently associated with IMT in addition to age, SBP, duration of diabetes, LDL-C, and VAT. While, calf circumference, smoking habits, and treatment medications were not associated with IMT (Table 4).

**Table 3:** Multiple stepwise regression analysis showing variables independently associated with insulin sensitivity index (Kitt)

	**Men (γ^2^ = 0.33)**	**Women (γ^2^ = 0.22)**
WC	−0.182^**^	−0.199^**^
FPG	−0.286^**^	−0.230^**^
TG	−0.110^**^	−0.139^**^
Insulin	−0.178^**^	−0.172^**^
HbA1c	−0.123^**^	−0.156^**^
VAT	−0.136^**^	−0.145^**^
Weight adjusted thigh circumference	0.128^**^	0.141^**^

* P < 0.05, **P <0.01.

WC, waist circumference; FPG, fasting plasma glucose; TG, triglyceride; HbA1c, hemoglobin A1c;VAT, visceral adipose thickness; Kitt, rate constant for plasma glucose disappearance.

**Table 4:** Multiple stepwise regression analysis showing variables independently associated with IMT

	**Men (γ^2^ = 0.18)**	**Women (γ^2^ = 0.23)**
Age	0.351^**^	0.347^**^
SBP	0.143^**^	0.151^**^
Duration of diabetes	0.094^**^	0.067^**^
LDL-C	0.108^**^	0.085^**^
VAT	0.073^**^	0.103^**^
Weight adjusted thigh circumference	−0.092^*^	−0.124^**^

* P < 0.05, **P <0.01.

SBP, systolic blood pressure; LDL-C, low-density lipoprotein cholesterol; VAT, visceral adipose thickness; IMT, intima media thickness.

According to multivariate logistic regression analysis, the OR of carotid atherosclerosis according to the quartiles of thigh circumference were showed in Table 5. However, the association of quartiles of calf circumference with carotid atherosclerosis was not significant (data not shown). The OR for carotid atherosclerosis increased according to the decreased thigh circumference based on an unadjusted model. Adjustments for age, duration of diabetes, smoking status, BMI, blood pressure, lipid, kitt, WC, VAT and treatment medications, the association between carotid atherosclerosis and thigh circumference remained significant. The results of regression analysis indicated that thigh circumference was independently correlated with insulin resistance and carotid atherosclerosis.

**Table 5:** The Odds ratios (95% CI) for carotid atherosclerosis according to thigh circumference quartiles

		**OR (95% CI)**	**Adjusted OR^a^ (95% CI)**
Men	Quartile 1	1.59* (1.00-2.35)	1.46* (0.98-2.27)
	Quartile 2	1.36* (0.98-1.91)	1.35* (0.99-1.93)
	Quartile 3	1.33* (0.95-1.88)	1.30* (0.93-1.64)
	Quartile 4	1	1
Women	Quartile 1	2.37** (1.57-3.55)	1.86** (1.16-3.05)
	Quartile 2	1.79** (1.17-2.73)	1.55** (0.95-2.32)
	Quartile 3	1.41* (0.98-2.13)	1.33* (0.82-1.89)
	Quartile 4	1	1

* P < 0.05, ** P <0.01.

^a^ Adjusted for age, duration of diabetes, smoking status, BMI, blood pressure, lipid, kitt, WC, VAT and treatment medications.

## Discussion

This study found that calf and thigh circumference was significantly correlated with insulin resistance and IMT, and thigh circumference was independently associated with insulin resistance and IMT in patients with type 2 diabetes. Furthermore, thigh circumference was an independent determinant factor for carotid atherosclerosis, even after adjusting for other cardiovascular risk factors.

There is growing evidence that peripheral adipocity and muscle mass are of major importance in determining insulin resistance [1,7,9,10]. Computed tomography (CT) is an accurate method for measuring body composition parameters including leg muscle mass and magnetic resonance spectroscopy is a powerful method for assessing lipid content in muscle [34-37]. However, the cost of CT or magnetic resonance spectroscopy is high, therefore CT and magnetic resonance spectroscopy are not suitable as a screening test. In contrast, thigh and calf circumferences are crude indices of muscle mass and peripheral subcutaneous fat as compared with CT and magnetic resonance spectroscopy, because thigh circumference and calf circumference reflect body muscle mass and peripheral subcutaneous fat [11,12], these measurements are noninvasive and easily applicable and they are more suitable as screening tools.

This study of 4,427 patients measured thigh and calf circumference and insulin resistance and demonstrated that insulin resistance was significantly correlated with thigh and calf circumference in patients with type 2 diabetes. The correlations were stronger with thigh circumference than calf circumference. Additionally, we found that thigh circumference was an important independent factor associated with insulin resistance in patients with type 2 diabetes. M. Ochi et al. recently reported that thigh muscle area did not appear to be significantly correlated with insulin resistance in elderly Japanese men [34]. In another study by Lee et al., thigh circumference was significantly associated with insulin resistance in peri- and postmenopausal women [13]. Taken together, these results suggest that thigh circumference, may be useful indicators of insulin resistance in Korean patients with type 2 diabetes.

Few studies have evaluated the relationship between body composition parameters such as thigh and calf circumference and atherosclerosis. Calf circumference is associated with carotid plaques in elderly subjects [23], thigh muscle area is related to ankle brachial pressure index (ABI) in elderly men [34], and reduced thigh muscle mass area is independently related to IMT in hemodialysis patients [24]. Recent studies reported that peripheral adiposity is associated with several markers of atherosclerosis such as aortic calcification, coronary angiography score, and arterial stiffness [38-40]. In the present study, calf circumference was correlated with IMT in women but not in men, and the association between calf circumference and IMT in women disappeared after regression analysis. But we found that decreased thigh circumference quartiles were also associated with increasing IMT in men and women, and thigh circumference was independently associated with IMT and carotid atherosclerosis. The increased odds ratio for carotid atherosclerosis was independent of general and abdominal obesity and cardiovascular risk factors, such as blood pressure and lipid. This results of the present study corroborate previous findings that low thigh circumference is associated with an increased risk of developing heart disease and premature death [12]. Although the mechanism underlying the relationship of thigh circumference and atherosclerosis is unknown, it was explained that less muscle mass and increased peripheral subcutaneous fat are associated with atherosclerotic markers [37-40].

Several limitations of our study need to be considered. First, based on its cross-sectional study design, the present findings are inherently limited in the ability to eliminate causal relationships between thigh and calf circumference and carotid IMT. Since some of the study population had several risk factors including hypertension, and dyslipidemia, the authors could not eliminate the possible effect of underlying diseases and medications used for these diseases on the present findings. Further prospective population-based studies are needed to investigate the mechanisms in order to answer these questions. Second, we did not measure thigh muscle area and did not consider muscle quality, such as muscle strength. Muscle strength has been shown to be more relevant to functional alteration than muscle mass in several studies [41]. Third, more detailed characteristics such as muscular lipid content assessed by CT, biopsy or magnetic resonance spectroscopy were not measured in this study. Kim et al. reported that altered lipid partitioning in skeletal muscle might be important in the development of insulin resistance and atherosclerosis [42]. Further studies employing imaging devices such as CT and MRI to measure calf and thigh muscle and lipid content could improve our understanding of the roles played by calf and thigh muscle in association with IMT as well as assess any differences by gender.

## Conclusions

In conclusion, calf and thigh circumference were correlated with insulin resistance and IMT, and thigh circumference was independently associated with insulin resistance and IMT in patients with type 2 diabetes. These results suggest that thigh circumference may be a new anthropometric marker of insulin resistance and carotid atherosclerosis.

## Abbreviations

SBP: Systolic blood pressure; DBP: Diastolic blood pressure; BMI: Body mass index; WC: Waist circumference; FPG: Fasting plasma glucose; TC: Total cholesterol; TG: Triglyceride; LDL-C: Low-density lipoprotein cholesterol; HDL-C: High-density lipoprotein cholesterol; HbA1c: Hemoglobin A1c; VAT: Visceral adipose thickness; IMT: Intima media thickness.

## Competing interests

The authors declare that they have no competing interests.

## Authors’ contributions

Study design, data analysis and manuscript writing: JSP; data collection and analysis: MHC; study design and conduct: CWA; Manuscript revision: KRK, KBH. All authors read and approved the manuscript.

## Supplementary Material

Additional file 1 **Figure S1.** The relationship between height, body weight, and thigh and calf circumference in men; P <0.01. **Figure S2**. The relationship between height, body weight, and thigh and calf circumference in women; P <0.01. Click here for file
