# Supplementary material for: The association of insulin resistance and carotid atherosclerosis with thigh and calf circumference in patients with type 2 diabetes
Source: Cardiovasc Diabetol. 2012 Jun 8;11:62. doi: 10.1186/1475-2840-11-62 (PMC3444381; doi:10.1186/1475-2840-11-62)
Supplement: Additional file 1 — Figure S1. The relationship between height, body weight, and thigh and calf circumference in men; P <0.01. Figure S2. The relationship between height, body weight, and thigh and calf circumference in women; P <0.01. [file 1475-2840-11-62-S1.docx]

r= 0.701

r= 0.729

r= 0.327

r= 0.280

Figure S1. The relationship between height, body weight, and thigh and calf circumference in men; P <0.01.

r= 0.171

r= 0.368

r= 0.744

r= 0.722

Figure S2. The relationship between height, body weight, and thigh and calf circumference in women; P <0.01.
